# Diagnostic Value of Conventional and Digital Radiography for Detection of Cavitated and Non-Cavitated Proximal Caries

**Published:** 2017-01

**Authors:** Mahdieh Dehghani, Rasool Barzegari, Hosein Tabatabai, Sahar Ghanea

**Affiliations:** 1 Assistant Professor, Department of Oral and Maxillofacial Radiology, Faculty of Dentistry, Shahid Sadoughi University of Medical Sciences, Yazd, Iran; 2 Dentist, Yazd, Iran; 3 Associate Professor, Department of Oral Pathology, Social Determinants of Oral Health Research Center, School of Dentistry, Shahid Sadoughi University of Medical Sciences, Yazd, Iran; 4 Assistant Professor, Department of Oral and Maxillofacial Radiology, Faculty of Dentistry, Rafsanjan University of Medical Sciences, Rafsanjan, Iran

**Keywords:** Dental Caries, Diagnostic Imaging, Radiography, Dental, Digital

## Abstract

**Objectives:**

This study aimed to assess the diagnostic value of conventional and digital radiography for detection of cavitated and non-cavitated proximal caries.

**Materials and Methods:**

Fifty extracted human premolars and molars were mounted in a silicone block. Charge-coupled device (CCD) and photostimulable phosphor plate (PSP) receptors and intra-oral films were exposed with 60 and 70 kVp with parallel technique. Two observers interpreted the radiographs twice with a two-week interval using a 5-point scale. Teeth were then serially sectioned in mesiodistal direction and evaluated under a stereomicroscope (gold standard). Sensitivity, specificity, positive predictive value, negative predictive value and accuracy were calculated.

**Results:**

Sensitivity of all three receptors for detection of enamel lesions was low (5.5–44.4%) but it was higher for dentin lesions (42.8–62.8%); PSP with 70 kVp and 0.03s exposure time had the highest sensitivity for enamel lesions, but the difference among receptors was not statistically significant (P>0.05). Sensitivity of all three receptors for detection of non-cavitated lesions was lower than that for cavitated lesions; PSP with 60 kVp and 0.07s exposure time had higher sensitivity and lower patient radiation dose for detection of cavitated and non-cavitated lesions, but the difference was not significant (P>0.05).

**Conclusions:**

Digital radiography using PSP receptor with 70 kVp is recommended to detect initial enamel caries. For detection of non-cavitated and cavitated dentin caries, PSP with 60 kVp is more appropriate. Change in kVp did not affect the diagnostic accuracy for detection of caries, and type of receptor was a more important factor.

## INTRODUCTION

Proximal carious lesions commonly occur and can be detected by noticing discoloration or coarseness at the site as well as radiography. Although discoloration and proximal surface coarseness may indicate caries, detection of carious lesion by direct observation is difficult, if not impossible. Thus, radiography is very important for detection of proximal caries [1]. Radiography is 88% more efficient for detection of proximal caries compared to direct observation [2].

Early detection of enamel lesions is very important for preventive treatment [3]. Studies showed that the depth of carious lesions can be dependent on the formation of cavities. For example, when a radiolucent lesion is detected in the internal half of dentin, it is more likely to form a cavity compared to radiolucencies in the enamel, but when a radiolucency is detected in the external half of dentin, it is difficult to determine whether the lesion is cavitated or not [4–6]. Insufficient processing of conventional images may affect interpretation. The time consuming processing of conventional radiographs, lower patient exposure to ionizing radiation in digital radiography and the possibility of changing the contrast and density after exposure in digital radiography have resulted in increasing popularity of digital compared to conventional radiography [7, 8]. Taking a radiograph with different exposure settings can affect the absorbed dose. Using higher voltage (kVp) decreases amperage (mAs) and absorbed dose, although it may also decrease contrast [2, 9]. In digital systems, it is important to create a constant pixel value in different exposure settings, which is dependent on an appropriate signal to noise ratio (SNR) [10]. Decreasing voltage (kVp) along with increased amperage (mAs) increases SNR, but simultaneously increases patient radiation dose, which is against as low as reasonably achievable or ALARA rule. On the contrary, increasing voltage (kVp) along with decreased amperage (mAs) decreases patient radiation dose but with lower SNR, primary noise may appear on the image and decrease contrast [11]. It is a wrong belief that digital images can always be modified after exposure. Different factors such as selected parameters, appropriate positioning of patient and the technician’s skills affect the quality of images [12]. Post-processing can improve the visibility of under or over-exposed images, but cannot correct the errors due to inappropriate patient positioning, and insufficient intrinsic contrast because of inappropriate primary exposure (which leads to lower SNR) [11].

Today, 60 and 70 kVp (instead of 50) are more commonly used [3]. Digital receiver panel or film in a given kVp/time has been previously evaluated, but studies on the effect of different voltage (kVp) conditions on detection of caries especially enamel lesions are limited. Thus, this study aimed to assess the accuracy of conventional and digital radiography for detection of proximal enamel and dentin carious lesions. The change in diagnostic accuracy after changing the voltage (kVp) was evaluated as well.

## MATERIALS AND METHODS

This was a cross-sectional study on extracted teeth. Fifty extracted human teeth (34 premolars and 16 molars) with sound surfaces or with cavitated or non-cavitated caries in their proximal surfaces were selected. Teeth were cleaned by a prophylaxis disc before mounting. Then, for disinfection, the teeth were stored in 5% chloramine T at 50°C for one week. The teeth were mounted in silicone putty blocks.

Each block contained one canine, two premolars and two molars, which were mounted in the silicon putty to the level of their cementoenamel junction simulating their anatomical positioning in the mouth. Their proximal surfaces were in contact with each other. The E-speed intra-oral films were exposed with a Minray dental X ray unit (Soredex, Tuusula, Finland). The photostimulable phosphor (PSP) plate (Soredex, Tuusula, Finland) with 40μm (super) pixel size, 14-bit grey scale, 12.5 lp mm^−1^ spatial resolution, and charge-coupled device (CCD; RH2 CNS Industries, Cinisello Balsamo, Italy) digital receptors (34×26 mm, a pixel size of <20 μm and a theoretical resolution of <28 lp mm^−1^) with standard parallel technique and a focus-receptor distance of 30 cm and XCP film holders were used for digital radiography. A 17 mm-thick acrylic plate was placed between the tube nd teeth for simulation of soft tissue.

Each block was irradiated with two different exposure settings: 1. Kodak E-speed film (Eastman Kodak, Rochester, NY, USA) with 60 kVp (7mA, exposure time: 0.2s) and 70 kVp (7mA, exposure time: 0.16s); 2. PSP and CCD digital receptors with 60 kVp (exposure time: 0.07s) and 70 kVp (exposure time: 0.03s). The exposure times were selected according to the guideline on the control panel. Radiographs were processed by a digital processor (Velopex, London, England) with processing solution (Jahan, Tehran, Iran). The PSP plates were read by Digora Optime scanner and then assessed using the software. The CCD and PSP plates were observed on a monitor (Sync Master; Samsung, Seoul, South Korea) in a quiet room with controlled light under similar conditions.

A dentomaxillofacial radiologist and a restorative dentist interpreted the conventional and digital radiographs twice with a two-week interval by a x2 magnifying glass. The mean of data obtained by the observers was used. The results of radiographic interpretations were reported according to a five-point scale used by Bottenberg et al [3]. The teeth were then serially sectioned in mesiodistal direction into 900 μm thick slices after removing from blocks using 820 μm thick saw in a cutting machine (T201A; Mecatome, Presi, France). The teeth were sectioned into four to six slices according to their mesiodistal dimension. The teeth were evaluated by a stereomicroscope (Optima Zoom; Digisystem Laboratory Instruments Inc., Taiwan) at x25 magnification. Opaque white demineralization and brown discoloration were considered as caries. In proximal surface of each tooth, first cavitation in the enamel was assessed and then the section with the deepest caries was scored by a pathologist according to the following scoring system and was considered as the standard: 0: Without caries, 1: Caries in the external half of enamel; 2: Caries extending to cementoenamel junction; 3: Caries in the outer half of dentin; 4: Caries extending to the inner half of dentin. The teeth with deep root caries were excluded from the study and substituted with other teeth. To compare the sensitivity and specificity of different receptors, receiving operating characteristic curves were drawn and cut-off point=1 was considered. The area under the curve (AZ) was calculated to compare the findings. Data were analyzed using SPSS version 17 (SPSS Inc., IL, USA) and Kappa statistic (Z test).

## RESULTS

According to the histological findings, 47 surfaces were free from caries, 18 had superficial caries in the enamel (scales 1 and 2), and 35 surfaces had deep caries (scales 3 and 4). In total, 60% of teeth with superficial or deep caries were not cavitated and the remaining were cavitated. For all imaging methods and different exposure settings, the sensitivity for detection of enamel caries (scales 1 and 2) with or without cavitation was low (5.5–44.4%; Table 1).

**Table 1:** Comparison of diagnostic value of conventional and digital radiography for detection of lesions limited to enamel and dentin according to kVp (%)

**Receptor**	**Diagnostic value (%)**									

	**Enamel**					**Dentin**				

	**Sensitivity**	**Specificity**	**Positive predictive value**	**Negative predictive value**	**Accuracy**	**Sensitivity**	**Specificity**	**Positive predictive value**	**Negative predictive value**	**Accuracy**
**PSP with 70 kVp**	44.4	75.6	28.5	86.11	70.0	54.2	84.6	65.5	77.4	74.0
**PSP with 60 kVp**	27.7	78.0	21.7	83.1	69.0	62.8	80.0	62.8	80.0	74.0
**Film with 70 kVp**	16.6	82.9	17.6	81.9	71.0	42.8	90.7	71.4	74.6	74.0
**Film with 60 kVp**	16.6	79.2	15.0	81.2	68.0	51.4	90.7	75.0	77.6	77.0
**CCD with 70 kVp**	5.5	82.9	6.0	80.0	69.0	45.2	73.8	48.4	71.6	64.0

The sensitivity of PSP receptor with 70 kVp for enamel caries was higher than others, but the difference was not statistically significant (P>0.05; Table 2). Intra-oral film with 70 kVp had the highest specificity and accuracy but without significant difference (P>0.05; Table 2). The sensitivity for dentin caries (scales 3 and 4) with or without cavitation was moderate (42.8–62.8%, Table 1). The highest sensitivity was observed in PSP receptor with 60 kVp, but this difference was not statistically significant (P>0.05, Table 2). Intra-oral film with 60 kVp had the highest specificity and accuracy but without significant difference (only the film was superior to CCD) (P>0.05, Table 2). Since all cavitated lesions were obviously carious, it was impossible to calculate sensitivity, specificity, positive predictive value and negative predictive value for these lesions (Table 3). In non-cavitated lesions, all diagnostic parameters were higher in lower kVp (Tables 1–3) and the highest sensitivity belonged to PSP receptor with 60 kVp, although its difference with intra-oral film with 60 kVp was not statistically significant (P=0.03), but difference with others was statistically significant (P=0.04, Table 4). Intra-oral film with 60 kVp had the highest specificity and accuracy in comparison with the two other receptors with significant differences (P<0.05, Table 4). In cavitated lesions in all scales, Z test failed to show a statistically significant difference between film, CCD and PSP with different exposure settings (Table 4) and sensitivity of all receptors in different exposure settings was almost equal. The sensitivity of three receptors in similar exposure settings was higher in cavitated lesions than non-cavitated lesions (Table 2). When statistical parameters were assessed according to the type of tooth (premolar and molar), it was found that in all exposure settings, the sensitivity of all three receptors was higher in premolars than molars, but the specificity was higher in molars (Table 3). Thus, the highest sensitivity, specificity and accuracy were observed in premolars with PSP receptors and 60 kVp, molars with intra-oral film in both kVp settings, and premolars with PSP receptor and 60 kVp (Table 5).

**Table 2:** Z-value for sensitivity and specificity of conventional and digital radiography for detection of lesions limited to enamel and dentin

**Receptor**	**Diagnostic value**			

	**Enamel**		**Dentin**	

	**Sensitivity**	**Specificity**	**Sensitivity**	**Specificity**
**Film with 60 kVp versus 70 kVp**	0.00	0.59	0.72	0.00
**PSP in 60 kVp versus 70 kVp**	1.04	1.70	0.73	0.69
**PSP in 60 kVp versus film in 70 kVp**	1.35	0.19	0.96	1.76
**PSP in 70 kVp versus film in 70 kVp**	1.81	1.16	0.51	1.07
**CCD in 60 kVp versus 70 kVp**	1.52	0.00	0.00	0.19
**CCD in 60 kVp versus film 60 kVp**	1.06	0.92	0.48	2.70
**CCD in 70 kVp versus film in 70 kVp**	1.61	0.00	0.75	2.56
**CCD in 60 kVp versus PSP in 60 kVp**	1.79	0.72	1.57	0.96
**CCD in 70 versus PSP in 70 kVp**	2.70	1.16	0.72	1.52

**Table 3:** Comparison of diagnostic value of conventional and digital radiography for detection of cavitated and non-cavitated lesions according to kVp (%)

**Receptor**	**Diagnostic value (%)**						

	**Non-cavitated lesions**					**Cavitated lesions**

	**Sensitivity**	**Specificity**	**Positive predictive value**	**Negative predictive value**	**Accuracy**	**Sensitivity**
**Film in 60 kVp**	71.9	93.6	88.46	81.01	84.81	85.7
**Film in 70 kVp**	56.3	93.6	85.71	75.86	78.48	81.0
**PSP in 60 kVp**	84.4	72.3	67.5	87.17	77.21	85.7
**PSP in 70 kVp**	81.3	72.3	66.6	85	75.94	85.7
**CCD in 60 kVp**	62.5	59.6	51.28	70	60.75	85.7
**CCD in 70 kVp**	56.3	72.3	58.06	70.83	65.82	81.0

**Table 4:** Z-value for sensitivity and specificity of conventional and digital radiography for detection of non-cavitated and cavitated lesions

**Receptor**	**Diagnostic value (%)**		

	**Non-cavitated**		**Cavitated**

	**Sensitivity**	**Specificity**	**Sensitivity**
**Film with 60 kVp versus 70 kVp**	1.31	0.0	0.41
**PSP with 60 kVp versus 70 kVp**	0.33	0.0	0.0
**PSP with 60 kVp versus Film with 60 kVp**	1.21	2.76	0.0
**PSP with 70 kVp versus Film with 70 kVp**	2.17	2.76	0.41
**PSP with 60 kVp versus Film with 70 kVp**	2.46	3.03	0.41
**PSP with 70 kVp versus Film with 60 kVp**	0.89	3.03	0.0
**CCD with 60 kVp versus 70 kVp**	1.06	1.31	0.41
**CCD with 60 kVp versus Film with 60 kVp**	0.85	4.25	0.0
**CCD with 70 kVp versus Film with 70 kVp**	0.0	3.03	0.0
**CCD with 60 kVp versus PSP with 60 kVp**	1.98	1.31	0.0
**CCD with 70 kVp versus PSP with 70 kVp**	2.27	3.03	0.41

**Table 5:** Comparison of diagnostic value of conventional and digital radiography in detection of cavitated and non-cavitated lesions according to type of tooth (%)

**Receptor**	**Diagnostic value (%)**										

	**Premolar**					**Molar**										

	**Sensitivity**	**Specificity**	**Positive predictive value**	**Negative predictive value**	**Accuracy**	**Sensitivity**	**Specificity**	**Positive predictive value**	**Negative predictive value**	**Accuracy**
**Film with 60 kVp**	86.76	85.0	89.28	91.9	80.6	81.25	62.5	100.0	100.0	72.7
**Film with 70 kVp**	80.88	77.27	87.5	91.9	67.7	75.0	55.55	100.0	100.0	63.6
**PSP with 60 KVP**	78.12	66.66	82.60	60.0	86.4	79.41	84.84	74.28	75.7	83.9
**PSP with 70 kVp**	75.0	81.25	69.44	70.3	80.6	84.37	72.72	90.47	80.0	86.4
**CCD with 60 kVp**	64.70	68.57	60.60	64.9	64.5	68.75	50.0	75.0	40.0	81.8
**CCD with 70 kVp**	72.05	73.68	70.0	75.7	67.7	62.5	42.85	77.77	60.0	63.6

The sensitivity for maxillary teeth in all receptors was higher than mandibular teeth, but the specificity was higher for mandibular teeth (Table 6). The highest sensitivity, specificity and accuracy were observed with PSP receptors and 60 kVp in the maxilla, intra-oral film receptors with 70 kVp in the mandible, and intra-oral film receptors with 60 kVp in the maxilla (Table 6).

**Table 6:** Comparison of diagnostic value of conventional and digital radiography for detection of cavitated and non-cavitated lesions according to the jaw (%)

**Receptor**	**Diagnostic value (%)**										

	**Mandible**					**Maxilla**										

	**Sensitivity**	**Specificity**	**value Positive predictive value**	**Negative predictive**	**Accuracy**	**Sensitivity**	**Specificity**	**Positive predictive value**	**Negative predictive value**	**Accuracy**
**Film with 60 kVp**	80.76	74.28	94.11	96.3	64.0	89.58	85.71	92.59	90.8	89.3
**Film with 70 kVp**	76.92	69.23	100.0	100.0	52.0	81.25	73.91	88.0	85.0	78.6
**PSP with 60 kVp**	75.0	76.92	73.07	74.1	76.0	83.33	85.5	81.25	70.0	92.9
**PSP with 70 kVp**	78.84	76.66	81.81	85.2	72.0	77.08	84.61	74.28	55.0	92.6
**CCD with 60 kVp**	61.53	64.0	59.25	59.3	64.0	77.08	84.61	74.28	55.0	92.6
**CCD with 70 kVp**	65.38	65.51	65.21	70.4	60.0	72.91	65.21	80.0	75.0	71.4

In final assessment of diagnostic parameters in all teeth (cavitated and non-cavitated), it was found that by increasing the kVp, sensitivity decreased in the three types of receptors (Table 7), although the difference was not statistically significant (P>0.05, Table 8), but the specificity was not different. We could not find a statistically significant difference between receptors regarding sensitivity in 60 kVp (P>0.05), but the difference was statistically significant with 70 kVp (P=0.01). The Z test showed a statistically significant difference between cavitated or non-cavitated groups regarding specificity in both kVp (Z ≥ 2.76 for both kVp; Table 8).

**Table 7:** Comparison of diagnostic value of conventional and digital radiography for detection of proximal lesions (%)

**Receptor**	**kVp**	**Diagnostic value (%)**						

		**Sensitivity**	**Specificity**	**Positive predictive value**	**Negative predictive value**	**Accuracy**
**Conventional**	60	77.4	93.6	93.18	75.57	85
	70	66	93.6	92.1	70.97	79
**PSP**	60	84.9	72.3	77.58	80.95	79
	70	83.0	72.3	77.19	79.06	78
**CCD**	60	71.70	59.6	66.66	65.11	64
	70	66.0	72.3	72.9	65.38	69

**Table 8:** Z-value for sensitivity and specificity of conventional and digital radiography for detection of lesions

**Receptor**	**Diagnostic value (%)**	

	**Sensitivity**	**Specificity**
**Film with 60 kVp versus 70 kVp**	1.3	0.0
**PSP with 60 kVp versus 70 kVp**	0.26	0.0
**PSP with 60 kVp versus film with 60 kVp**	0.99	2.76
**PSP with 70 kVp versus film with 70 kVp**	2.21	2.76
**PSP with 60 kVp versus film with 70 kVp**	2.27	2.76
**PSP with 70 kVp versus film with 60 kVp**	0.72	2.76
**CCD with 60 kVp versus 70 kVp**	0.7	1.31
**CCD with 60 kVp versus film with 60 kVp**	0.7	4.25
**CCD with 70 kVp versus film with 70 kVp**	0.0	3.03
**CCD with 60 kVp versus PSP with 60 kVp**	1.65	1.31
**CCD with 70 kVp versus PSP with 70 kVp**	2.12	0.0

Finally, it was found that intra-oral film with 60 kVp, PSP with 60 kVp, and intra-oral film with both 60 and 70 kVp had the highest accuracy, sensitivity and specificity, respectively (Table 7). The voltage (kVp) did not have a significant effect on sensitivity and specificity of intra-oral film and digital receptors for detection of caries, but the type of receptor had a significant effect on sensitivity and specificity (P=0.03; Table 8).

## DISCUSSION

In this study, inter and intra-observer agreement was high (78.75 and 89.25, respectively), probably due to the fact that observers were expert specialists in this field. In this study, different imaging methods (film, CCD and PSP) were compared regarding accuracy for detection of caries with different exposure settings. In our study, all methods had a low sensitivity for enamel lesions regardless of the type of receptor, which was consistent with the results of the study conducted by Botenberg et al [3]. They found sensitivity between 6% and 40% for F and D films and CCD and CMOS receptors for detection of caries in the enamel. Castro et al, [13] found that none of the receptors of E-speed film and CMOS had an acceptable accuracy for detection of enamel lesions (AZ = 61–65% for enamel compared to AZ = 84–88% for dentin). Pontual et al, [14] found that all imaging methods (PSP and intra-oral film) had a low sensitivity (14–16%) for detection of enamel caries. These results are predictable because enamel lesions have an irregular shape and low contrast.

Increasing the depth of lesion (when it is confined to the enamel) does not increase its visibility on radiographs [14]; this finding has been confirmed in other studies as well [15–17]. Haiter-Neto et al, [18] found that in three dimensional (Accuitomo, NewTom 6, 9 and 12 inch field of view), two dimensional (Insight film) and digital (Digora) systems, the ability to detect lesions in the enamel was low (13–21%). These systems had a higher sensitivity for detection of caries in dentin (31–58%). In our study, all three receptors had a higher sensitivity for detection of lesions in dentin. In a study conducted by Peker et al, [19] intra-oral radiography and digital radiovisiography were not able to detect carious lesions in the enamel. Senel et al, [20] showed that the sensitivity of CCD and PSP receptors, film and CBCT for detection of enamel lesions was low (7.3–18.7%), and among these, PSP had higher sensitivity, which was in agreement with the results of our study. The PSP plate allows for post-processing and enhancement of desired areas; thus, it can improve the diagnostic accuracy and reduce disagreement between observers [21].

An interesting finding of the current study was the higher sensitivity of PSP in comparison with intra-oral film for detection of enamel lesions although it had lower spatial resolution in higher voltage (kVp). This advantage is probably due to its software features. in our study, PSP with 70 kVp and 0.3s exposure time was probably the best choice for detection of enamel lesions compared to other exposure conditions regardless of the type of receptor; although the difference was not statistically significant, probably due to the fact that X-ray can penetrate into the enamel and its intensity is not so high to cause burn out. Botenberg et al, [3] found that for detection of enamel caries, voltage (kVp) change in all three receptors (CMOS, CCD, and intra-oral film) did not affect the accuracy of radiography. By increasing the tube potential and decreasing the time of irradiation, patent radiation dose decreases, but the image contrast decreases as well. In the current study, increasing the voltage from 60 to 70 kVp decreased the diagnostic parameters for detection of non-cavitated lesions, but in cavitated caries, this difference was not significant. In non-cavitated carious lesions, if the lesion is small, its detection is strongly affected by image contrast due to lower contrast between sound and carious tissue and higher voltage (kVp) leads to decreased contrast and may create a long gray-scale image [22]. This effect is obvious in non-cavitated lesions, but in cavitated lesions, voltage (kVp) change is not as effective due to the presence of cavity and higher contrast between tissues. In our study, we used an anatomical scale (discrete) for categorizing the lesions in comparison to the confidence scale (continuous). Other studies have also used this scale [23, 24]. The assessment of sensitivity, specificity and accuracy of diagnostic tests showed that PSP receptors had a higher sensitivity, and for detection of carious lesions, PSP was superior to intra-oral film and CCD; although intra-oral film was more accurate with less false positive results.

In order to observe ALARA rule, if there is clinical signs or discoloration in a tooth, especially with a sign of cavity, it is recommended to use PSP with 60 kVp, because it has a higher sensitivity and lower exposure time in comparison to intra-oral film. Considering the small difference between 60 and 70 kVp regarding exposure time (0.4 s) in both receptors, and lower absorbed dose in 60 kVp according to the following formula: dose=(mR/mAs)(kVp)^2^, and no significant difference between PSP and intra-oral film in detection of cavitated caries and lower exposure time in PSP in relation to intra-oral film and other advantages of digital over analogue systems, when there is positive clinical signs and a cavitated lesion, it is recommended to use PSP receptor with 60 kVp. In studies conducted for assessment of caries, usually a pilot study is done on a limited number of teeth and the appropriate exposure setting is determined as such.

Considering the small size of the samples in the pilot study and obvious changes in exposure conditions, it seems that the results of these kinds of studies cannot be used for detection of different depths of caries [3]. Arnold [25] assessed the effect of change in exposure settings with D and E films and found that changes in kVp insignificantly affected the ability to detect carious lesions, which was consistent with our results. Kaeppler et al, [26] failed to show significant difference between 60 and 90 kVp in the ability to detect carious lesions created by diamond bur. In their study by increasing the voltage (kVp), exposure time and subsequently the absorbed dose significantly decreased. They artificially created carious lesions; thus, the sensitivity of receptors in their study was higher than that in our study, but it seems that the results cannot be generalized to real carious lesions, because X-ray tubes used in dentistry work with 50–70 kVp and higher kVp is not routinely used [22]. Hintze and Wenzel [15] found that the accuracy of radiography with Ekta-speed plus film is higher than Dixi (CCD-based, Planmeca, Finland) for detection of proximal caries and both methods were better than RVG, but Haiter-Neto et al, [18] found that diagnostic parameters for detection of proximal caries with Insight film and Digora digital system (CCD-based) were similar. Findings of Castro et al, [13] were consistent with the results of our study. They found that intra-oral film was slightly more accurate for detection of caries in enamel and dentin compared to digital system with CMOS sensor; although the difference was not statistically significant. The accuracy of conventional radiography with Ekta-speed Kodak film was similar to digital radiography (RVG, Trophy) and tomography in the study of Peker et al, [19] which was consistent with our results. Senel et al, [20] also found results similar to ours; they did not find a significant difference between film, CCD and PSP receptors in detection of proximal caries, although intra-oral film had a higher sensitivity than CCD and PSP. Syriopoulos et al, [27] could not find a significant difference between intra-oral film, CCD and PSP in detection of proximal caries and they concluded that the experience of the observer was much more important.

In assessment of the lesions, diagnostic value was higher for cavitated lesions, which was more apparent in E-film receptor than PSP and was consistent with the results of the study conducted by Bottenberg et al [3]. They found that diagnostic value was 10% higher for cavitated lesions.

For early detection of initial carious lesions, radiography is preferred to clinical examination [28]. Fluorescent-based techniques have been introduced as substitutes for radiography for detection of initial enamel lesions. These methods have shown a high sensitivity in studies, although they are not used routinely [29–32].

Although radiography has a low sensitivity for detection of enamel lesions, it is still the method of choice. In the recent years, due to advances in PSP receptors, and the lower patient radiation dose in this method, this receptor has been frequently used in intra-oral radiography. It is recommended to design in-vivo studies and studies for assessment of the diagnostic accuracy of CBCT for detection of enamel lesions.

## CONCLUSION

According to the results of this study, radiography using PSP receptor with 70 kVp is recommended for detection of initial enamel caries. For detection of non-cavitated and cavitated caries in dentin, PSP with 60 kVp was more appropriate. Change in voltage (kVp) did not affect the diagnostic accuracy for detection of caries, and the type of receptor was a more important factor in this regard.
